# Predicting retention time in hydrophilic interaction liquid chromatography mass spectrometry and its use for peak annotation in metabolomics

**DOI:** 10.1007/s11306-014-0727-x

**Published:** 2014-09-07

**Authors:** Mingshu Cao, Karl Fraser, Jan Huege, Tom Featonby, Susanne Rasmussen, Chris Jones

**Affiliations:** 1 AgResearch Grasslands Research Centre, Palmerston North, 4442 New Zealand; 2 Massey University, Institute of Agriculture and Environment, Palmerston North, New Zealand

**Keywords:** QSRR, LCMS, Metabolomics, Peak annotation, Metabolite identification, *Lolium perenne*

## Abstract

Liquid chromatography coupled to mass spectrometry (LCMS) is widely used in metabolomics due to its sensitivity, reproducibility, speed and versatility. Metabolites are detected as peaks which are characterised by mass-over-charge ratio (*m/z*) and retention time (rt), and one of the most critical but also the most challenging tasks in metabolomics is to annotate the large number of peaks detected in biological samples. Accurate *m/z* measurements enable the prediction of molecular formulae which provide clues to the chemical identity of peaks, but often a number of metabolites have identical molecular formulae. Chromatographic behaviour, reflecting the physicochemical properties of metabolites, should also provide structural information. However, the variation in rt between analytical runs, and the complicating factors underlying the observed time shifts, make the use of such information for peak annotation a non-trivial task. To this end, we conducted Quantitative Structure–Retention Relationship (QSRR) modelling between the calculated molecular descriptors (MDs) and the experimental retention times (rts) of 93 authentic compounds analysed using hydrophilic interaction liquid chromatography (HILIC) coupled to high resolution MS. A predictive QSRR model based on Random Forests algorithm outperformed a Multiple Linear Regression based model, and achieved a high correlation between predicted rts and experimental rts (Pearson’s correlation coefficient = 0.97), with mean and median absolute error of 0.52 min and 0.34 min (corresponding to 5.1 and 3.2 % error), respectively. We demonstrate that rt prediction with the precision achieved enables the systematic utilisation of rts for annotating unknown peaks detected in a metabolomics study. The application of the QSRR model with the strategy we outlined enhanced the peak annotation process by reducing the number of false positives resulting from database queries by matching accurate mass alone, and enriching the reference library. The predicted rts were validated using either authentic compounds or ion fragmentation patterns.

**Electronic supplementary material:**

The online version of this article (doi:10.1007/s11306-014-0727-x) contains supplementary material, which is available to authorized users.

## Introduction

Metabolomics aims to provide a systems-level measurement of all the metabolites in biological samples. Multiple analytical platforms must be employed to achieve this goal because of the enormous physicochemical diversity of small molecules and their broad dynamic range in cellular concentration. Due to its high sensitivity, high sample throughput, accurate detection of mass-over-charge ratio (*m/z*) and compact instrumentation, mass spectrometry coupled to chromatography has become the dominant analytical platform in metabolomics. Signals detected from these platforms, such as liquid chromatography mass spectrometry (LCMS), are described as the pair of *m/z* and retention time (rt). Thousands of peaks can be routinely detected and quantified from crude extracts of biological samples, largely thanks to the advances in mass spectrometry and the progress in developing data analysis software. For example, soft ionization methods such as electrospray ionization (ESI) enable direct analysis of polar and thermally labile biomolecules in their intact form (Fenn et al. 1990), while among data processing tools, XCMS (Smith et al. 2006) and MZmine (Pluskal et al. 2010) are tools of choice in the public domain.

A common practice in LCMS based metabolomics is to first identify significant peaks (mass-over-charge ratio *m/z* denoted hereafter as mz for describing peaks) of biological relevance via computational and statistical ranking approaches and then to carry out structural inference on a few selected peaks. Inferences on peak identity are made by matching the measured *m/z* and rt of the top ranking peaks (mz/rt) with that of authentic compounds usually maintained in an in-house reference library. However, such practices run into serious limitations in metabolomics as there are far fewer reference standards than the number of peaks that can be detected from biological samples (Dunn et al. 2013; Kind and Fiehn 2010; Wishart 2011). The number of unknown peaks compromises the systems approach to interpret the quantitative variation and to address biological problems (Patti et al. 2012), thus peak annotation on a large scale is an imperative task in metabolomics.

Highly accurate *m/z* measurement enables the prediction of the elemental composition of unknown peaks. This accurate mass measurement, together with additional mass spectral features such as isotopic patterns, is often utilized for chemical annotation of detected peaks (Kind and Fiehn 2006; Draper et al. 2009; Iijima et al. 2008). However, compounds with the same exact mass but different structures cannot be differentiated by accurate mass alone. For instance, the amino acids leucine (Leu) and isoleucine (Ile) have the same mass of 131.0946 Da (monoisotopic mass) but different structures. To characterize these two amino acids the information collected from either multi-stage MS or chromatography must be exploited. Chromatographic retention time, reflecting the chemical properties (hydrophobicity, polarity, molecular shape etc.) of detected peaks, can provide further information to infer the chemical class and possible chemical structure of peaks (Kuehnbaum and Britz-McKibbin 2013). Nevertheless, rt values measured by LC–MS on the same compound often vary considerably depending on the experimental conditions such as column packing, flow rate and mobile phase composition. Experimental rt values are therefore difficult to harness for the annotation of unknown peaks and for information sharing between research groups. Continuing improvements on resolution and reproducibility in chromatography, which promise to provide reliable measurement of rts, would permit the systematic use of rts for the structural inference of peaks. Peak annotation based on accurate mass has been extensively investigated, and research has recently been called upon to utilize the chromatographic side of information for compound identification (Spagou et al. 2010; Boswell et al. 2011; Hall et al. 2013). One of the critical steps towards the systematic utilization of rt for peak annotation is to associate peak rt with the structural and/or physiochemical properties of the measured chemical components.

Molecular descriptors (MDs) define the structural and physiochemical properties of molecules by assigning numeric values through mathematical and statistical approaches (Todeschini and Consonni 2009). Structural information such as type of atoms and bonds, number of rings, charge and stereochemical configuration can be encoded in MDs. The Wiener index, for example, is a structural descriptor that can describe the topology of molecules by counting the number of bonds between pairs of atoms and summing up the distance between all pairs. LogP (octanol/water partition coefficient in the logarithmic scale), a widely used MD, is a physiochemical descriptor, which measures the lipophilicity of molecules (Mannhold et al. 2009). MDs have often been used for Quantitative Structure and Properties Relationship (QSPR) and Quantitative Structure and Activities Relationship (QSAR) modelling with the purpose of predicting the biological properties and activities of compounds (Jónsdóttir et al. 2005). MDs are also used to model chromatographic retention time of new compounds in the absence of standard candidates via Quantitative Structure–Retention Relationship (QSRR) modelling (Héberger 2007). QSRR modelling has usually been carried out on a particular class of compounds measured in respective analytical platforms (Sarkhosh et al. 2012; Tyrkkö et al. 2012; Meek 1980). Only recently has QSRR modelling found an application in metabolomics (Creek et al. 2011; Hagiwara et al. 2010) because there is a demand to assign chemical identities to many unknown peaks through improved utilization of retention time, along with mass spectral features.

To establish a QSRR model we need to: (1) represent molecular structures in a computable format; (2) calculate MDs from the structural representation; (3) collect experimental rts of a number of authentic compounds based on a particular analytical platform; and finally (4) establish the model. In this study, we used an open source Java library CDK (Chemistry Development Kit) (Steinbeck et al. 2003) to compute MDs from canonical SMILES (Simplified Molecular Input Line Entry System)—a popular structural representation of molecules (Weininger 1988; O’Boyle 2012); The experimental rts for 116 authentic compounds (standards) were manually recorded from a hydrophilic interaction LC coupled to high resolution ESI MS (HILIC-MS) platform (Fraser et al. 2012). Modelling of rts as a function of the theoretically or experimentally derived MDs was often established by Multiple Linear Regression (MLR) and machine learning algorithms such as artificial neural network (ANN), regression tree and support vector machine (SVM) (Jónsdóttir et al. 2005; Héberger 2007; Put et al. 2003). We employed MLR and Random Forests (RF) (Breiman 2001a) methods to establish a predictive QSRR model because the two methods represent two different approaches to modelling, i.e. data modelling and algorithmic modelling (Breiman 2001b). MLR is a widely used statistical method in QSRR whereas RF is suitable for handling a mixture of continuous and discrete variables, which is the case for MDs.

Here, we established a QSRR model for a HILIC-MS analytical platform, and evaluated the effectiveness of this model to annotate peaks (mz/rt) detected in perennial ryegrass (*Lolium perenne*) samples. We demonstrate that model-based rt prediction provides additional information for peak annotation, which cannot be ascertained by matching accurate mass alone. A general strategy is outlined to iteratively improve the model, to validate the prediction and to enrich the LC–ESI–MS-based library for peak annotation. The promises and limitations of such approaches are also discussed.

## Materials and methods

### Sampling and analytical methods

Both the 116 authentic compounds (Sigma-Aldrich, Auckland, NZ, see Table S1) and the plant extracts (*L. perenne* leaf blade tissue) were analysed using HILIC coupled to high resolution orbitrap Exactive MS (Thermo, Waltham, MA, USA). The 116 authentic compounds, covering a wide range of polarity, were initially selected for building a reference library. The retention times of these compounds were recorded manually and employed for building QSRR models in this study. Eight plant samples were taken from a large metabolomics study on the drought responses of perennial ryegrass (*L. perenne*), a major forage grass in the temperate regions of the world. These eight samples, representing a single genotype selected from a genetically segregating population, were subjected to drought challenge (*n* = 4) and irrigated control conditions (*n* = 4) during the growing season, and were all harvested at the same developmental stage. More sample information relevant to this investigation can be found in the supplementary materials (Data S1). This subset of samples was selected to illustrate the application of QSRR modelling to annotate unknown but statistically significant peaks differentiating between the two treatment groups.

Plant sample preparation, extraction and experimental setups for the HILIC-MS were the same as those previously described (Fraser et al. 2012). Briefly, samples were extracted with 50:50 acetonitrile–water (v/v) and separated on a Merck polymeric bead based ZIC-pHILIC column (100 × 2.1 mm^2^, 5 µm, zwitterionic stationary phase) using a mixture of acetonitrile-formic acid (solvent A) and water–ammonium formate (solvent B, pH 6.3) as the mobile phases. Chromatography was performed at 25 °C with a gradient elution programme that held at 97 % A (0–1 min), 97–70 % A (1–12 min), 70–10 % A (12–14.5 min), 10 % A (14.5–17 min), returned to 97 % A (17–18.5 min) and allowed to equilibrate for a further 5.5 min prior to the next injection. Data were collected in profile data acquisition mode (with positive ESI) over a mass range of *m/z* (60–1200) at a mass resolution setting of 25,000 (at *m/z* 400). With the predefined resolving power (R), the mass window (∆m) can be theoretically defined by m/R, i.e. 400/25,000 = 0.016, which is equivalent to 20 ppm (∆m) for mass = 200.

Peak detection on the raw data collected from perennial ryegrass samples was carried out using MZmine (Pluskal et al. 2010) with the noise level being set to 5,000 (5e3) for exact mass detection. Chromatograms (for each mass that can be detected continuously over scans) were built by time span = 0.2 min, the minimum peak height = 2e4 and *m/z* tolerance with parts per million (ppm) = 20; Chromatogram deconvolution was performed using the “noise amplitude” approach with minimum peak height = 5e4 and duration time 0.6 min; Peaks were de-isotoped using the built-in functions (*m/z* = 0.01 and rt = 0.1 min) and peak alignment across samples was performed by the Join Aligner algorithm implemented in MZmine. As a result, 2,859 peaks (mz/rt) were detected in the eight samples. Local peak detection from extracted ion chromatograms (XIC) was conducted using a wavelet-based approach (Du et al. 2006). A univariate non-parametric test (Kruskal test) was used to identify peaks that were significantly different between the drought-stressed and control groups. Among the significant peaks a few were selected for the detailed discussion on peak annotation.

### Calculation and data pre-processing of molecular descriptors

Canonical SMILES representations for the 116 standard compounds and plant metabolites were obtained from the PubChem database (http://pubchem.ncbi.nlm.nih.gov/) if available, otherwise generated using chemical structural editors, JChemPaint (http://jchempaint.github.io/) or using the PubChem online chemical structure sketcher (http://pubchem.ncbi.nlm.nih.gov/edit2/index.html).

The models were built in this study based on theoretical MDs instead of experimental physicochemical properties. MDs were calculated from SMILES structural representation using the R package “rcdk” (Guha 2007), which is based on CDK—a Java library for chemo-informatics (Steinbeck et al. 2003). A total of 346 MDs were calculated (using rcdk 3.2) for each standard compound (in its neutral form). These MDs represent various physical and chemical properties of the compounds, such as hydrophobicity, polarity and topology. The calculated MDs comprise many different data types including continuous and discrete values, and redundant representations of the same properties. MDs that represent protein structures and properties were discarded. MDs with >90 % missing values or with constant values were also removed. If a group of MDs belonged to the same class (for example, SPC.4, SPC.5 and SPC.6—Chi path cluster descriptors which describe molecular connectivity) and they were highly correlated (Pearson’s correlation coefficient, *r* > 0.9), only one MD (in this case, SPC.4 of the lowest order) was retained. Detailed description of all MDs can be referred to in the monograph (Todeschini and Consonni 2009) or an online version of CDK API (http://qsar.sourceforge.net/dicts/qsar-descriptors/index.xhtml). LogP has been found to be the most important parameter in QSRR modelling, but it can vary because many algorithms can be used to compute LogP (Mannhold et al. 2009). We used XLogP computed by CDK, whereby the implementation is based on atom types (Wang et al. 1997; Wang et al. 2000). XLogP from PubChem, however, is the implementation of a modified version based on XLogP3 (Cheng et al. 2007). XLogP3 data were obtained from PubChem for the 116 reference compounds to compare with the CDK-based XLogP. They were largely correlated (*r* = 0.80) although discrepancies can be seen (Fig. S1). CDK XLogP was chosen for QSRR modelling in this study, as in the case of a metabolite being not available from PubChem, its structure (in SMILES) can be obtained using structure editors, such as JChemPaint.

### Modelling approaches

After data cleaning of the calculated MDs those retained MDs were subjected to wrapper-based feature selection, where a subset of MDs was selected by the prediction model itself. MLR model selection was undertaken by an exhaustive search for the best subset with four different model selection criteria, i.e. Mallow’s Cp, Akaike information criterion (AIC), Bayesian information criterion (BIC) and adjusted R^2^ using an R package “leaps” (http://CRAN.R-project.org/package=leaps). Feature selection and predictive modelling by Random Forests (RF) algorithms were conducted using the R package “randomForest” (Liaw and Wiener 2002). To ensure feature stability, RF (with 500 trees in each forest) were built 100 times, and those features with >50 % occurrence at the respective ranking positions were selected to establish the final predictive model. Model training and resampling-based evaluation were carried out with utility functions from the “caret” package (http://CRAN.R-project.org/package=caret). All data processing, statistical analysis, model building and evaluation were conducted in the R statistical computing environment (R Development Core Team 2013).

### Databases

PubChem (http://pubchem.ncbi.nlm.nih.gov) was used to query a list of candidate compounds for rt prediction. Canonical SMILES of compounds were downloaded from PubChem for the standards used in this study and the testing compounds used for validation of predicted rts. Other online databases such as METLIN (http://metlin.scripps.edu) and Chebi (http://www.ebi.ac.uk/chebi) were used for cross references. We also used the PlantCyc (www.plantcyc.org) compound database, which includes 3,202 unique metabolites in the version of 2013-07-24. The PlantCyc compound database was downloaded onto a local computer to allow automatic calculation and searching in a batch mode. Monoisotopic masses were calculated for all the entries with valid chemical formulae (e.g. excluding the arbitrary representations for polymers) in their neutral form by custom R scripts.

## Results and discussion

### QSRR model construction and evaluation

**Fig. 1:**
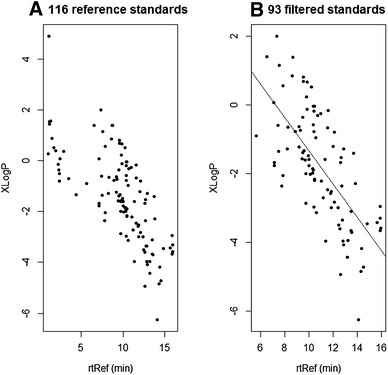
**a** Overall negative correlation was observed between the experimental retention time of the reference compounds (rtRef) and XLogP (CDK-based calculation) for the 116 reference compounds which were used for the HILIC-based LCMS library construction; **b** Compounds with rt < 5 min and duplicated stereoisomers were not retained, leaving 93 compounds for the modelling process. A significant correlation between rtRef and XLogP was shown (*r* = −0.69, *p* value < 2.0e-14)

For the 116 standard compounds a negative correlation between XLogP and the experimental retention times (rts) was clearly discernible (Fig. 1a), indicating that the more hydrophilic molecules have longer retention time in the HILIC column. Positive and negative LogP suggest either a hydrophobic or a hydrophilic nature of the molecule. The magnitude of the LogP value is indicative of the strength of affinity for water. Sixteen of the analysed standard compounds with rt < 5 min were excluded in the subsequent modelling process to ensure models were constructed with only those compounds that fully interacted with the chromatographic system. The small rt differences between stereoisomers such as l-isoleucine (9.70 min) and d-isoleucine (9.87 min) are due to measurement error and beyond the resolution of the chromatographic systems being employed. Therefore, seven redundant isomers (see supplementary material “QSRR_peakAnnotation_R.pdf”) with the same structural representation in SMILES were also excluded, leaving a total of 93 reference compounds. An overall correlation between XLogP and rt is shown in Fig. 1b (*r* = −0.69, *p* value < 2.0e-14, *n* = 93). However, XLogP alone may not have enough power to predict rt. For example GABA (γ-aminobutyric acid) and xanthine have similar calculated XLogP values (−0.67 and −0.65) but the rt of GABA was recorded as 11.55 min and xanthine 8.29 min (Table S1).

**Fig. 2:**
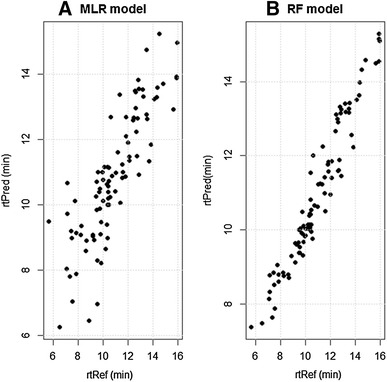
Correlation between the predicted retention time (rtPred, min) and the experimental retention time (rtRef, min) for the 93 reference compounds by the established models **a** Multiple Linear Regression (MLR) (*r* = 0.85), and **b** Random Forest (RF) model (*r* = 0.97)

In addition to XLogP we performed a feature selection to determine if we could identify a set of MDs that could better explain the recorded rts of these standards. By exhaustive searching (branch-and-bound algorithm implemented in the “leaps” package) we conducted a model selection to find the best subset of MDs to predict rt in MLR. MLR models were evaluated based on four criteria including Mallow’s Cp and Akaike information criterion (AIC), Bayesian information criterion (BIC) and adjusted R^2^. Eleven MDs (model size) were selected as the best subset according to these four criteria (see Fig. S2). These 11 MDs (bpol, nHBDon, ATSc1, ATSp1, VP.0, fragC, VABC, VAdjMat, WPATH, WPOL, XLogP, see Data S2 for the details on the descriptors) were then utilized to construct the final predictive MLR model. A repeated 10-fold cross validation was applied to estimate prediction performance of the model. As a result, the mean accuracy of the model has an adjusted R^2^ of 0.64. The predicted rt (rtPred) correlated with the measured rts of the reference compounds (rtRef) with *r* = 0.85 (Fig. 2a). The absolute prediction error (|rtPred—rtRef|) has a mean of 0.95 and a median of 0.76 min, which is equivalent to 9.4 and 6.7 % in terms of percent relative error, respectively. Six MDs, XLogP, bpol, nHBDon, VP.0, fragC and WPATH were determined to be the most significant MDs (*p* values < 0.001) for predicting rt.

Because the relationship between MDs and observed rts of compounds may be complex, alternative approaches to MLR, which may offer a more robust method to model the relationship and provide better prediction accuracy, were explored. RF algorithm was employed here to construct a collection of regression trees for the rt prediction. By growing a forest of trees and the injection of some randomness RF is robust against overfitting (Breiman 2001a) in comparison to a single regression tree model (Put et al. 2003). Because of the randomness implemented in the algorithm RF were built 100 times (500 trees in each forest) and only those MDs consistently ranked at the top were selected to build the predictive model. A subset comprising of XLogP, BCUTp.1h, TopoPSA, and nHBAcc were then elected. These 4 MDs were used to build the final predictive model via a repeated 10-fold cross-validation. As a result, the predicted results (rtPred) are correlated with the RTs of reference compounds (rtRef) with *r* = 0.97, suggesting RF outperforms MLR (*r* = 0.85) (Fig. 2). The unsigned prediction error of the RF model was a mean = 0.52 min and median = 0.34 min, which is 5.1 and 3.2 %, respectively, when expressed as percent relative error.

XLogP was found to be the most contributing predictor in both MLR and RF models. This is in agreement with the results reported previously (Creek et al. 2011). Their model, also based on HILIC-MS, revealed that LogD (similar to LogP, but pH-dependent) was the most predictive variable out of six other calculated properties including charge, the number of rotatable bonds, the number of phosphate groups and the number of hydrogen bond donors divided by molecular weight (HBD/MW). The QSRR model reported by Hagiwara et al. (Hagiwara et al. 2010) was constructed using both MLR and support vector regression (SVR) based on XLogP, TPSA and Complexity that were downloaded from PubChem, and a custom computed MD, i.e. solvent-accessible surface area (ASA), to model the interaction between the column and the compound. The usefulness of rt prediction in assisting compound identification without the use of reference standards was also demonstrated in these two studies which helped inform this research project.

The retention mechanism in HILIC is complex but polarity has been reported to be the main factor, along with others such as electrostatics (Cubbon et al. 2010). As identified from our results the partition coefficient (XLogP), polarity related MDs, i.e. BCUTp.1h (describing atomic static polarizability) and TopoPSA (topological polar surface area) and nHBAcc were determined to be the main features to model compound separation behaviour in the ZIC-pHILIC column. Therefore, XLogP and the two polarity-related MDs can be readily explained. The descriptor nHBAcc, which calculates the number of hydrogen bond acceptors and contributed to our model prediction, might explain the interaction between solutes and the stationary phase via hydrogen bonds. The interpretation of some MDs selected by MLR (e.g. WPATH, a Wiener numbers descriptor) is beyond our knowledge, as this is sometimes the case in QSRR/QSPR modelling. In this situation, the predictive power and the usefulness in the application to actual problems can still be a strong motive to establish a model (Todeschini and Consonni 2008; Héberger 2007).

The mean unsigned error was 0.95 min from the MLR model, and 0.52 min from the RF model. The median error for the RF model-based prediction was 0.34 min, and this prediction accuracy suggests it approximates the chromatographic resolution in the current system. The RF model provides improved results compared to those previously reported, where the mean and median absolute errors were 1.12 and 0.84 min (Tyrkkö et al. 2012; Creek et al. 2011). In addition to that, we recruited MDs for QSRR modelling via a systematic, unbiased feature selection process, rather than based solely on prior knowledge.

The correlation between rtPred and rtRef and the mean squared error (MSE) are useful metrics to assess the overall goodness of fit of QSRR models, and it is of practical interest to examine the distribution of the residuals, i.e. the distribution of the differences between the predictions and the experimental observations. The standard deviation (sd) of prediction errors (rtPred-rtRef) is 0.68 (Fig. S3). In the following discussion we chose to use rtPred ± 0.68, i.e. rtPred within a 0.68 min window around the observed peak retention time (rtPeak), as a criterion for judging whether a prediction matches the experimental rt. This is approximately 11.3 % of error for a compound eluting at 6 min and 5.7 % of error for a compound eluting at 12 min—a narrower error range than that reported by Creek et al. (2011), who used within 35 % of the predicted retention times to achieve improved metabolite identification by removing 40 % of the false identifications that occurred with identification by accurate mass alone.

We acknowledge that this criterion is suggestive. The evaluation of error distribution should provide a rigorous test for the confidence of the prediction. The validity of this criterion can be tested by future studies when more authentic compounds or annotated metabolites become available (the current evaluation was based on 93 compounds). An iterative process is thus proposed in the Sect. (3.3) to improve the model resolution and thus prediction accuracy. Confident rt prediction can also be compromised by the measurement error of peak rt, which is due to time shifting among samples. Variation of rtPeak should therefore be carefully examined in order to make a robust inference in the process of peak annotation.

Besides these statistical considerations, it should be noted that no attempt was made to differentiate stereoisomers (*Z/E* or *R/S*) here as they tend to co-elute under the experimental conditions used in our study. For example, the experimental rt for authentic l-isoleucine and d-isoleucine was 9.70 and 9.87 min, and 12.80 and 12.85 min for l-glutamic acid and d-glutamic acid, respectively (see Table S1). These subtle differences in rt are probably due to measurement error and beyond the resolution of the chromatographic systems being employed. Therefore, only one stereoisomer was retained for building the models, and stereochemistry is not specified in the following discussion on peak annotation.

### Application of the QSRR model to peak annotation

We have identified below three scenarios for the application of the established QSRR model for peak annotation in a metabolomics study.

**Fig. 3:**
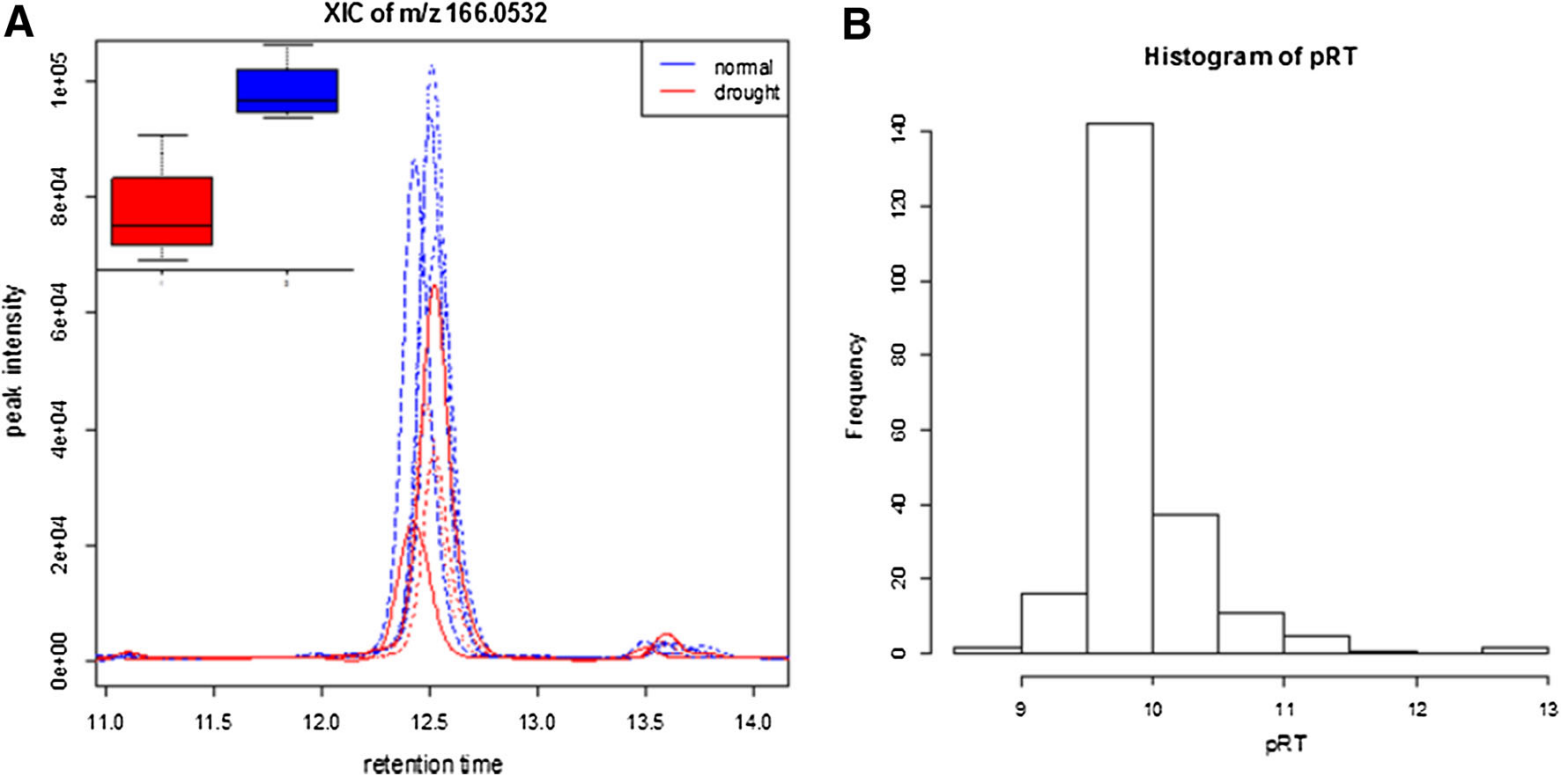
The smoothed XIC of *m/z* 166.0532 ± 20 ppm from the eight samples. The *boxplot* shown (**a**) was based on the normalised peak heights from wavelet-based peak detection. Histogram (**b**) of the predicted retention time (pRT) of 216 PubChem compounds with the same chemical formula of C_5_H_11_NO_3_S

In the first scenario we show that our model can help reduce false positives considerably. Peak 166.0532/12.50 (mz/rt) was one of the significant peaks (Kruskal test, *p* value <0.05) identified in *L. perenne* blade tissue in response to drought (Fig. 3a). Assessment of the mass spectra indicated that this is a singly charged species ([M+H]^+^) with *m/z* of 166.0530. We undertook chemical formula prediction of mass 165.0457 (in its neutral form). When C, H, N, O, S, and P were included in the element search list and a few empirical rules such as H/C ratios and isotopic ratio filtering, were implemented (Kind and Fiehn 2007), C_5_H_11_NO_3_S was the only candidate molecular formula for the accurate mass (see Data S3). However, a search of the formula in PubChem resulted in 269 compounds, preventing further annotation of this formula. The RF-based rt prediction model was therefore used to narrow down the candidates. After the disconnected SMILES forms such as “C1CCS(=O)(=O)C1.C(=O)N” (separated by a period ‘.’) and redundant SMILES were removed, 216 compounds remained for rt prediction. The prediction results are summarized in Fig. 3b, only two compounds, methionine sulfoxide (cid 847) and ethiin (cid 146416), with a predicted rt of 12.67 and 12.59 min, respectively, matched this peak at 12.50 min (±0.68). The two compounds are also recorded in the PlantCyc compound database suggesting their involvement in plant metabolism. We conducted an independent validation experiment (Method S1, Fig. S5) by spiking the authentic compound methionine sulfoxide (ethiin was not available for purchase) into a ryegrass extract, showing that the rt of the standard was 12.81 min (Data S3), thus enabling the peak of 166.0532/12.50 to be annotated as methionine sulfoxide or ethiin.

In the next scenario of the QSRR model application we show that the predicted rt can provide additional annotation information to a hypothetical metabolite, whose structure may not be available from public databases. Thesinine-rhamnoside (C_23_H_31_NO_7_, neutral mass = 433.2101) is a plant alkaloid known to occur in perennial ryegrass (Koulman et al. 2008). However, its structural information has not yet been deposited in any public databases. In order to predict the rt of this metabolite in the chromatographic system used here, we obtained its SMILES presentation “C12([H])CCCN1CCC2COC(=O)C=CC3=CC=C(C=C3)OC4OC(C)C(O)C(O)C4O” by JChemPaint structure editor, and computed MDs based on this structural representation. The MD predictors required by the RF model were computed as XLogP = 1.24, BCUTp.1h = 9.47, TopoPSA = 108.69 and nHBAcc = 8. Using our model the predicted rt of this molecule though the HILIC column is 8.90 min. We then examined the XIC of this metabolite ([M+H]^+^) of *m/z* 434.2173 (±20 ppm) from the eight samples, and observed that a peak eluted at 9.1 min in all of the samples (Fig. S8). The measured mass (*m/z* 434.2175) was within a deviation of 0.46 ppm from the theoretical calculation, and the accurate match between the predicted rt (8.90 min) and the experimental rt (9.1 min) of the chromatographic peaks allows the positive annotation of the peak as thesinine-rhamnoside. Further interrogation of the mass spectral data demonstrated that the experimental isotopic pattern matched the theoretical pattern (See Data S4). As no standard compound is available for us to validate the predicted rt a separate validation experiment was performed based on the MS^2^ fragmentation of the *m/z* 434.22 (Data S4—Fig. S7). The fragmentation pattern supported the identity of the peak (434.22/9.5) with the evidence of the presence of a typical fragment ion (*m/z* 288.16), due to the loss of a rhamnose residue (*m/z* 146.06) (Koulman et al. 2008).

Positive peak identification requires equivalent information collected from authentic or chemically synthesized compounds (Sumner et al. 2007). However, in metabolomics research the number of authentic compounds is limited and artificially synthesized compounds can be expensive or even impossible to obtain for metabolite identification (Wishart 2011; Zhu et al. 2013). QSRR predictive models can provide predicted rt, an information orthogonal to accurate mass for a putative identification. Therefore, it may be legitimate to deposit the predicted rt along with theoretical mass into the library to facilitate annotation. The expansion of the reference library (usually built upon a list of authentic compounds) by adding putative annotations provides an indispensable step to address metabolite identification problems in large scale metabolomics studies.

In the third scenario we discuss the use of rt prediction to annotate closely eluting peaks, which can be challenging. Peak 132.1023/8.9 was of interest as it increased in abundance under drought conditions (Kruskal test, *p* value <0.05) (Fig. S8). The rts of Leu (9.5) and Ile (9.7) were recorded in the library (Table S1), and it is tempting to annotate this peak as Leu based on the match in mass (1.5 ppm) and rt (0.6 min). However, there are five chromatographic peaks of *m/z* 132.1023 and baseline separation was not achieved for two of these (Fig. S8). The direct application of our current model (with a 0.68 min prediction window) is not useful in this kind of situations where the predicted rts for Leu (10.0) and Ile (9.9) are within 0.1 min. The clear-cut annotation of the peaks is consequently beyond the resolution of the current model, which is, in turn, reliant on the resolution of the chromatography employed. The rts recorded in the library were based on a mixture of standard compounds, and the question remains as to how the rts recorded from the mixture of pure compounds relate to the measured peaks (metabolites) occurring in the crude biological extracts. Therefore, spiking experiments were performed to confirm that the peaks at 9.57 and 9.86 min correspond to Leu and Ile, respectively (See Data S5). With that information peak 132.1023/8.9 was excluded from being Leu or Ile. We applied the same procedures (as that used in the first scenario) to search for other possible annotations of this peak. Based on its accurate mass the peak can be predicted with a formula of C_6_H_13_NO_2_, and 970 compounds were found in PubChem with this formula (distinct canonical SMILES). From the range of predicted rts 8.17–11.06 min only 18 compounds had a predicted rt in the range of 8.9 ± 0.3 min (Data S5). The predictions help again to narrow down the list, making identification more feasible. A search of the PlantCyc compound database based on accurate mass resulted in three metabolites (Leu, Ile and β-alanine betaine). The rt prediction for β-alanine betaine was 9.7 min, ruling it out as a possibility for peak 132.1023/8.9. Further evidence remains to be collected before this peak can be annotated. Usefulness of the QSRR model to avoid false annotation was further supported by the annotation of another statistically significant peak 287.0551/7.08. Without considering the eluting behaviour *m/z* 287.0551 could be annotated as kaempferol (K) in its protonated form ([M+H]^+^), which is a common flavonoid in *L. perenne*. However, the QSRR model suggests the protonated kaempferol elutes at 8.0 min. Further inspection of the peak 287.0551/7.08 indicates that it was an in-source fragment ion of a flavonoid glycoside (K-AcHex-Rha), demonstrating the ability of the QSRR model to assist identification and avoid false annotations.

### A general strategy

**Fig. 4:**
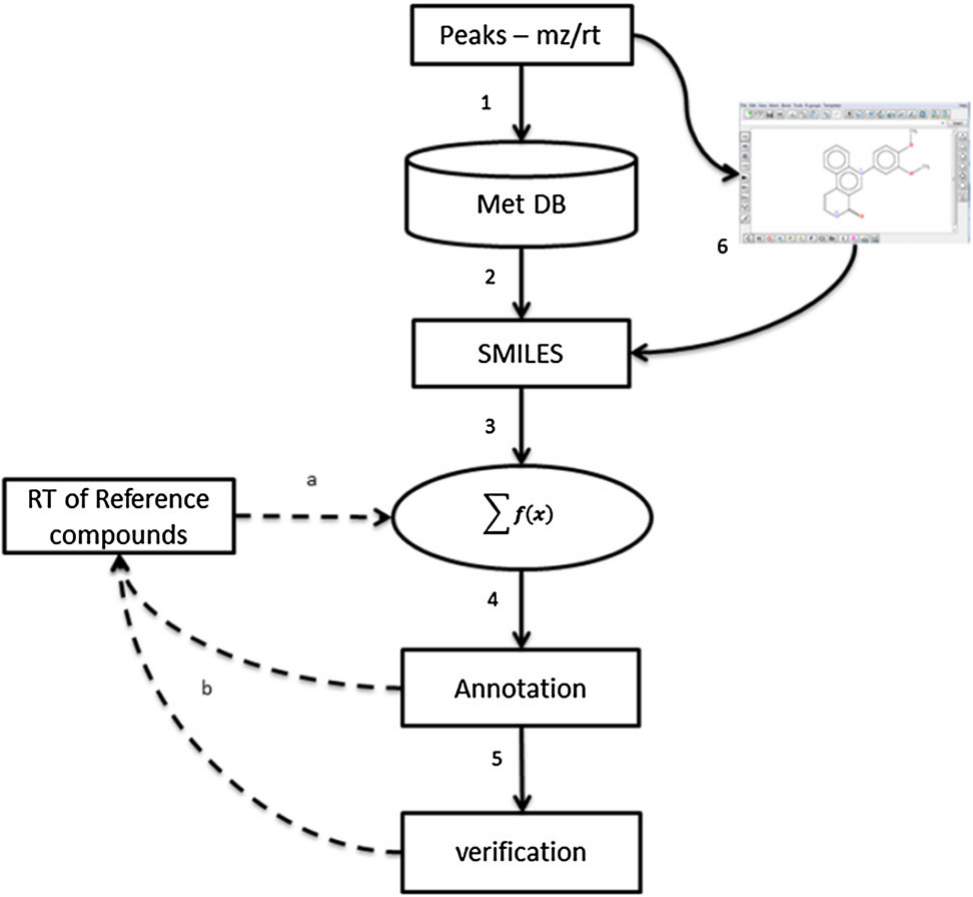
Diagram of the modelling process (literal: *a*, *b*) and the application of the established model for peak annotation (number: 1–6). *a* build a QSRR model based on experimental retention time (rt) of known compounds (a reference library); *b* update the model by incorporating the newly verified or putatively identified compounds. The model can be iteratively improved. *1* search databases with the measured accurate mass; *2* integrate and refine the query results from various resources and compute the structural presentation (SMILES) of the query list; *3* compute molecular descriptors and predict rt using the model; *4* annotate peaks by adding the predicted rt and its prediction accuracy; *5* verify the predicted rt with other evidence; *6* when no hits returned from database search by accurate mass, hypothetical compounds occurring in biological samples can be proposed and their structures can be sketched using a molecular editor to generate structural presentation

We have demonstrated the power of model-based rt prediction to assist the annotation of unknown peaks. Although there are areas for further improvement, particularly with regards to the extent to which prediction accuracy can be achieved given the relatively low resolution of the chromatographic systems, we anticipate that such a model-based rt prediction promises general applications on peak annotation in LCMS-based metabolomics studies. Hence, we have outlined a strategy for the modelling process and application of the predictive models (Fig. 4). Further to this, we provide recommendations for the practical use of such a strategy to improve peak annotation.

As shown in Fig. 4 a proof-of-concept QSRR model can be built first using the experimental rt of the authentic reference compounds, which should be selected to cover a wide range of retention times (Step a). Putatively annotated or verified peaks derived from the annotation process can be recruited into the list of reference compounds to update the model (Step b). The incorporation of the putatively identified compounds into the reference library not only helps improve the predictive power of the QSRR model but also expands the library for future identification, with the putatively identified metabolites tagged as “putative” in the library in contrast to the “authentic” standards. False positives may occur in such an expanded library but can be controlled as meta-information is maintained and corrected whenever supporting evidence becomes available. A simple database (a few interlinked tables) can be designed for more robust annotation (which is beyond the discussion of this paper).

A few quality control steps should be implemented to start the annotation process on a list of detected peaks (mz/rt). These should include the investigation of the XIC of the detected ion to check data quality and ion types (protonated/deprotonated, adduct ions, in-source fragments etc.) as suggested by (Zhu et al. 2013). Accurate monoisotopic masses (in neutral form) are used first to search public or in-house databases by a pre-defined mass error window, with a unit of accuracy that depends on the resolving power of the mass analyser employed (Step 1). This may return a list of named metabolites from the database. It remains debatable as to which database should be used in the first instance. As we demonstrated in this study it is computationally feasible to perform rt prediction on a large scale if structural formulae are readily available in the databases. More generalized databases certainly expand the list of compounds to be tested for rt prediction, which is necessary during investigation of novel leads. On the other hand, a specialised database (organism-specific if available) can help reduce the number of false positives. Structural formula in SMILES, SDF etc. can either be obtained from databases (Step 2) or generated from chemical structure editors (Step 6). In this paper, we have demonstrated modelling process and automatic calculation of MDs required by the model (Step 3). If the model-based rt prediction matches with rtPeak (within a defined error range) the peak can be putatively annotated (Step 4). Additional (or orthogonal) evidence such as fragmentation patterns can be used to validate these putative annotations along with comparing experimental structural features with that of authentic compounds (Step 5). This strategy enables the model to be updated iteratively by incorporating the putatively identified or verified compounds.

At present an enormous effort is required to compare the retention behaviours of the same metabolites among different chromatographic systems. Therefore, despite being challenging, the incorporation of chromatographic conditions in building a predictive QSRR model deserves continued research (Boswell et al. 2011). Comparative QSRR modelling among different chromatographic systems is necessary to study chromatographic behaviour of the same set of metabolites in different systems and to reveal their invariant structural features and physiochemical properties.

Although our methodology should be readily extendable to other chromatographic systems widely employed in metabolomics studies, different models must be developed for each chromatographic technique because of the different separation mechanism involved. Likewise, a specific set of MDs is likely to be recruited during the modelling process for different chromatographic systems. For example, we have discussed the annotation of peak 434.2175/9.0 as thesinine-rhamnoside, and there is only one chromatographic peak detected on the ZIC-pHILIC column (Data S4). But two isomers (*E/Z*) of thesinine-rhamnoside, known to be present in *L. perenne*, can be readily separated by reversed phase liquid chromatography (C18 column) (Koulman et al. 2008). This suggests that when conducting the QSRR modelling for the C18 column 3D MDs need to be recruited.

Even with a match on both accurate mass and retention time it may still not be sufficient to annotate the majority of peaks in LCMS-based metabolomics. Additional evidence such as MS^n^ fragmentation patterns need to be collected to increase the rigor of structural inference on the detected peaks (Cao et al. 2013), and integrated into a reference library or database for identification.

## Conclusions

We have established a QSRR model based on the RF algorithm for the prediction of retention time of compounds and achieved prediction accuracy at a level that can be readily employed for peak annotation in LCMS-based metabolomics. We have demonstrated that such model-based retention time prediction can reduce considerably the number of false positives that often arise from a query of accurate mass alone, and we have proposed a general strategy to incorporate QSRR modelling into the metabolite annotation process. We thus conclude that our approach allows the retention time to be harnessed and integrated into the peak annotation processes, and contributes to address the most challenging problems in metabolomics, that is to know the unknowns.

## Electronic supplementary material

Below is the link to the electronic supplementary material.

Supplementary material 1 (CSV 34 kb)

Supplementary material 2 (DOCX 424 kb)
