# Supplementary material for: Predicting retention time in hydrophilic interaction liquid chromatography mass spectrometry and its use for peak annotation in metabolomics
Source: Metabolomics. 2014 Sep 7;11(3):696–706. doi: 10.1007/s11306-014-0727-x (PMC4419193; doi:10.1007/s11306-014-0727-x)
Supplement: Supplementary file 2 — Supplementary material 2 (DOCX 424 kb) [file 11306_2014_727_MOESM2_ESM.docx]

Supplementary Materials

**Predicting retention time in hydrophilic interaction liquid chromatography mass spectrometry and its use for peak annotation**

Mingshu Cao*, Karl Fraser, Jan Huege, Tom Featonby, Susanne Rasmussen, Chris Jones

AgResearch Grasslands Research Centre, Palmerston North 4442, New Zealand

*Corresponding author

Email: [Mingshu.Cao@agresearch.co.nz](mailto:Mingshu.Cao@agresearch.co.nz)

Phone: +64 6 351 8163

**Table S1**. 116 authentic compounds with measured *m/z* (mz), retention time (rt) and PubChem ID (cid). Other information such as formula and canonical SMILES can be obtained from the online PubChem database by querying cid.

| **Compound** | **adduct** | **mz** | **rt** | **cid** |
| --- | --- | --- | --- | --- |
| L-Theanine | M+H+ | 175.1075 | 10.61 | 439378 |
| Pyridoxine | M+H+ | 170.0812 | 9.21 | 1054 |
| Beta-Penta acetyl glucose | M+NH4+ | 408.15 | 1.06 | 79064 |
| O-methyl threonine | M+H+ | 134.0812 | 10.41 | 2724875 |
| Indole-3-acetamide | M+H+ | 175.0866 | 2.48 | 397 |
| Isonipecotic acid | M+H+ | 130.0863 | 11 | 3773 |
| Diethanolamine | M+H+ | 106.0863 | 11.35 | 8113 |
| Dianisidine o- | M+H+ | 245.1285 | 1.46 | 8411 |
| Imidazole | M+H+ | 69.04473 | 10.36 | 795 |
| L-Glutamine | M+H+ | 147.0762 | 12.9 | 5961 |
| L-threonine | M+H+ | 120.0655 | 12.5 | 6288 |
| L-Serine | M+H+ | 106.0503 | 13.25 | 5951 |
| Hypoxanthine | M+H+ | 137.0458 | 7.88 | 790 |
| L-Homoserine | M+H+ | 120.0659 | 12.6 | 12647 |
| Xanthone | M+H+ | 197.0597 | 1.22 | 7020 |
| D-Tryptophan | M+H+ | 205.0969 | 10.1 | 6305 |
| L-Isoleucine | M+H+ | 132.1022 | 9.7 | 6306 |
| 5-methoxytryptamine | M+H+ | 191.1179 | 9.56 | 1833 |
| 7-Azaindole | M+H+ | 119.0604 | 1.74 | 9222 |
| 4-(dimethyl amino) cinnamaldehyde | M+H+ | 176.107 | 1.17 | 5284506 |
| D-Leucine | M+H+ | 132.1022 | 9.5 | 6106 |
| Alanine | M+H+ | 90.0555 | 12.1 | 5950 |
| Cinchonidine | M+H+ | 295.1805 | 8.69 | 101744 |
| D(+)turanose | 2M+Na+ | 707.2217 | 12.62 | 5460935 |
| L-cysteine hydrochloride hydrate | M+H+ | 122.027 | 11.88 | 23462 |
| Lactic acid | M+H+ | 91.03898 | 7.45 | 61503 |
| Colchicine | M+H+ | 400.1755 | 1.36 | 6167 |
| L-Arginine | M+H+ | 175.119 | 15.97 | 6322 |
| L-Proline | M+H+ | 116.0706 | 10.4 | 145742 |
| L-Lysine | M+H+ | 147.1128 | 15.9 | 5962 |
| L-Asparagine | M+H+ | 133.0609 | 13.2 | 6267 |
| Creatine Anhydrous | M+H+ | 132.0768 | 12.03 | 24892273 |
| Creatinine | M+H+ | 114.0662 | 9.87 | 588 |
| Folic Acid | M+H+ | 442.147 | 11.38 | 6037 |
| L-Glutamic acid | M+H+ | 148.0604 | 12.8 | 33032 |
| p-aminohippuric acid | M+H+ | 195.0764 | 5.67 | 2148 |
| DL-Aspartic acid | M+H+ | 134.0448 | 13.56 | 5960 |
| L-phenylalanine | M+H+ | 166.0863 | 9.43 | 6140 |
| D-valine | M+H+ | 118.0866 | 10.5 | 6287 |
| L-Histidine monohydrochloride | M+H+ | 156.0769 | 15.65 | 6274 |
| L-tyrosine | M+H+ | 182.0812 | 11.2 | 6057 |
| D-glutamic acid | M+H+ | 148.0601 | 12.85 | 23327 |
| Rutin Trihydrate | M+H+ | 611.1586 | 9.3 | 5280805 |
| D-kynurenine | M+H+ | 209.0916 | 9.76 | 1152206 |
| N,N-Dimethylbenzylamine | M+H+ | 136.1122 | 7.54 | 7681 |
| 4,8-Dihydroxyquinoline-2-carboxylic acid | M+H+ | 206.0441 | 9.54 | 5699 |
| N,N’-Dicyclohexylcarbodiimide | M+H+ | 207.1852 | 1.09 | 10868 |
| Morpholine | M+H+ | 88.076 | 10.37 | 8083 |
| 2-Amino-2-methyl-1,3-propandiol | M+H+ | 106.0867 | 12.69 | 1531 |
| Quinaldic acid | M+H+ | 174.0549 | 1.9 | 7124 |
| Methionine DL- | M+H+ | 150.0583 | 10.33 | 876 |
| Nicotinic acid | M+H+ | 124.0395 | 2.35 | 938 |
| Riboflavin | M+H+ | 377.1449 | 7.85 | 493570 |
| Phloridzin | M+H+ | 437.1431 | 7.09 | 6072 |
| Dihydroxyphenyl-analine | M+H+ | 198.076 | 12.73 | 6047 |
| Guanine | M+H+ | 152.0567 | 9.81 | 764 |
| Inosine monophosphate | M+H+ | 349.0543 | 13.84 | 8582 |
| Guanosine | M+H+ | 284.0984 | 10.6 | 6802 |
| Inosine | M+H+ | 269.0876 | 9.18 | 6021 |
| Cytosine | M+H+ | 112.0507 | 10.41 | 597 |
| Morin | M+H+ | 303.0497 | 6.55 | 5281670 |
| Xanthine | M+H+ | 153.0405 | 8.29 | 1188 |
| Glycocyamine | M+H+ | 118.0614 | 12.81 | 763 |
| Adenosine | M+H+ | 268.1042 | 7.78 | 60961 |
| 5-Amino valeric acid | M+H+ | 118.0867 | 10.72 | 138 |
| Naringin CHR | M+H+ | 581.186 | 7.51 | 442428 |
| Tryptamine | M+H+ | 161.1074 | 9.86 | 1150 |
| Diaveridine | M+H+ | 261.1342 | 8.91 | 21453 |
| O-phospho-L-threonine | M+H+ | 200.0319 | 14.52 | 3246323 |
| DL-Homocysteine | M+H+ | 136.0428 | 11.06 | 778 |
| D-Methionine | M+H+ | 150.0586 | 10.23 | 84815 |
| Cysteine | M+H+ | 122.0275 | 11.83 | 5862 |
| Cinchonine | M+H+ | 295.1796 | 8.75 | 90454 |
| Gamma-amino-n-butyric acid | M+H+ | 104.0708 | 11.55 | 119 |
| Adenine | M+H+ | 136.0618 | 8.22 | 190 |
| Dibenzylamine | M+H+ | 198.1272 | 7.37 | 7656 |
| Cacotheline | M+H+ | 428.1439 | 11.58 | 221125 |
| Theophylline | M+H+ | 181.0717 | 2.28 | 2153 |
| DL-2-6-Diaminopimelic acid | M+H+ | 191.1023 | 14.85 | 865 |
| Taurine | M+H+ | 126.0219 | 12.64 | 1123 |
| L-(+)-Norvaline | M+H+ | 118.0863 | 10.39 | 65098 |
| L-2-Aminoadipic acid | M+H+ | 162.0759 | 12.4 | 92136 |
| D-Isoleucine | M+H+ | 132.1018 | 9.87 | 76551 |
| 5-Methoxy-DL-tryptophan | M+H+ | 235.1072 | 9.74 | 119802 |
| L-tert-Leucine | M+H+ | 132.104 | 10.04 | 164608 |
| Trans-4-hydroxy-L-proline | M+H+ | 132.0675 | 12.13 | 5810 |
| L-a-Amino-n-butyric acid | M+H+ | 104.0725 | 11.56 | 6657 |
| DL-2-4-Diaminobutyric acid dihydrochloride | M+H+ | 119.0837 | 15.92 | 470 |
| L-Norleucine | M+H+ | 132.1042 | 9.62 | 21236 |
| D-Norvaline | M+H+ | 118.0884 | 10.46 | 439575 |
| 6-(γ-γ-Dimethylallylamino)purine | M+H+ | 204.1186 | 2.88 | 92180 |
| L-Ornithine | M+H+ | 133.0979 | 15.93 | 6262 |
| Glycylglycine | M+H+ | 133.0616 | 14.39 | 11163 |
| Thymine | M+H+ | 127.051 | 3.51 | 1135 |
| Urea | M+H+ | 61.0404 | 7.16 | 1176 |
| P-Aminobenzoic acid | M+H+ | 138.0553 | 2.26 | 978 |
| Ethanolamine | M+H+ | 62.0608 | 12.89 | 700 |
| Benzylamine | M+H+ | 108.0814 | 10.24 | 7504 |
| L-Alpha-Glycerophosphate | M+H+ | 173.0216 | 13.52 | 3707990 |
| N-Acetyl-D-galactosamine | M+H+ | 222.0978 | 9.94 | 35717 |
| Iminodiacetic acid | M+H+ | 134.0456 | 13.71 | 8897 |
| L-Phenyl-d5-alanine-2,3,3-d3 | M+H+ | 174.137 | 9.69 | 13000987 |
| Thymidine | M+H+ | 243.0984 | 4.41 | 5789 |
| Acetamide | M+H+ | 60.0451 | 2.38 | 178 |
| Thymidine monophosphate | M+H+ | 323.0647 | 11.86 | 9700 |
| Tyramine | M+H+ | 138.0918 | 10.46 | 5610 |
| Deoxyguanosine | M+H+ | 268.1047 | 9.48 | 638 |
| Pyridoxal-5'-phosphate | M+H+ | 248.0328 | 10.3 | 1051 |
| Nicotinamide adenine dinucleotide | M+H+ | 664.1188 | 14.14 | 5892 |
| Deoxycytidine | M+H+ | 228.0986 | 10.04 | 13711 |
| Cytidine | M+H+ | 224.0937 | 10.48 | 6175 |
| 2'-Deoxyadenosine | M+H+ | 252.1099 | 7.15 | 13730 |
| Catechin | M+H+ | 291.0872 | 8.63 | 9064 |
| Lactobionic acid | M+H+ | 359.1195 | 14.3 | 7314 |
| Lactose (Peak A) | M+H+ | 343.1241 | 13.49 | 6134 |
| N-Acetyl-D-glucosamine | M+H+ | 222.0965 | 9.96 | 24139 |

**Data S1.** Sample information of 8 perennial ryegrasses. These 8 samples were run in the same analytical batch.

*pkHP filename sampleId geno dry endo DM*

2 20110708_rg_vp+006 G6_DM4_227 6 1 0 DM4

31 20110708_rg_vp+039 G6_DM4_468 6 2 0 DM4

38 20110708_rg_vp+048 G6_DM4_639 6 1 0 DM4

46 20110708_rg_vp+058 G6_DM4_125 6 2 0 DM4

64 20110708_rg_vp+078 G6_DM4_856 6 2 0 DM4

89 20110708_rg_vp+107 G6_DM4_547 6 2 0 DM4

98 20110708_rg_vp+118 G6_DM4_302 6 1 0 DM4

100 20110708_rg_vp+120 G6_DM4_743 6 1 0 DM4

**Figure S1**. Calculated descriptor XLogP of 100 authentic compounds based on the different algorithms (PubChem and CDK) are correlated (r=0.8). Discrepancy between the two implementations is noticeable with the mean absolute difference of 0.78 and the range of [-2.94, 2.87]. This may suggest the evaluation of the different implementations of XLogP for QSRR modelling is necessary.

**Figure S2**. Model selection based on four criteria (Cp, AIC, BIC and adjusted R^2^). Multiple linear regression (MLR) were evaluated for searching the best subset (model size in terms of the number of parameters) of molecular descriptors (MDs). As shown here 11 MDs were then selected to build the predictive MLR model.

**Data S2.** Description of selected molecular descriptors discussed in the study

XLogP constitutional – describe hydrophobic/hydrophilic

BCUTp.1h hybrid – chemical diversity

TopoPSA topological and electronic –topological polar surface

nHBAcc electronic -- electrostatic

nHBDon electronic -- electrostatic

bpol electronic -- polarity

ATSc1, ATSp1 topological

VP.0, topological

fragC topological -- fragment complexity

VABC topological

VAdjMat topological

WPATH topological

WPOL topological

SPC.4, SPC.5, SPC.6 topological – describe molecular connectivity

Refer to the online version of CDK API for more details:

<http://qsar.sourceforge.net/dicts/qsar-descriptors/index.xhtml>

<http://pele.farmbio.uu.se/nightly/api/org/openscience/cdk/qsar/descriptors/molecular/>

**Figure S3**. The distribution of the prediction errors (RF model), i.e rtPred – rtRef based on the 93 authentic compounds (from Table 1) with standard deviation of 0.68. The standard derivation of the prediction errors based on MLR model is 1.24.

**Data S3.** Further evidences on the annotation and validation of peak 166.0532/12.50

Formula prediction of 166.0530 [M+H]^+^ using a custom R function “getFormula” resulted in 3 candidate chemical formula. Here was the output of “getFormula” function.

> getFormula(166.0530-1.007276, e="C50H120O50N10S4P3")

A reminder: please provide monosiotopic mass by taking care of adduct, Rule2

cformula massT ppm

[1,] "C7H8N3P" "165.045583886" "0.8"

[2,] ***"C5H11NO3S" "165.045964212" "1.5"***

[3,] "C5H13NOP2" "165.047237296" "9.2"

"C7H8N3P" and "C5H13NOP2" can be easily filtered out by checking their isotopic ratios.

**Figure S4.** Isotopic patterns of C_5_H_11_NO_3_S shown as the comparison of the theoretical distribution in green and the experimental isotopic ions (within 10 ppm) in black. Data were retrieved from data file 20110708_RG_VP+058.mzXML.

**Method S1.** Spiking experiments: Mass spectrometer analysis was performed on a Thermo Exactive mass spectrometer as outlined by Fraser et al. (2012)(see the reference in the main text). The spiking experiment was set up as such. The original ryegrass extract was analysed along with separate standard solutions of 100 ug/mL Methionine sulfoxide, 100 ug/mL D-Leucine and 100 ug/mL iso-Leucine. From the retention time correlation and original peak height in the ryegrass extract, three ryegrass extracts spiked with the three different standards above were prepared, with the aim of approximately doubling the targeted peak by spiking with each standard. This was achieved by first measuring the intensity of the peak in the standard, comparing it to the intensity observed in the ryegrass extract and spiking accordingly. This new set of ‘spiked’ samples was then run under the same conditions and compared to the original ryegrass extract to confirm identity of peaks.

**Figure S5.** Spiking experiment to validate the predicted retention time of Methionine sulfoxide. Ryegrass extract (plant no: G6-856) and the pure compound methionine sulfoxide with XIC plotting of *m/z* 166.05 peak presented as: upper – methionine sulfoxide; middle – ryegrass plant extract; lower – plant extract with the pure compound methionine sulfoxide spiked.

**Table S2**. METLIN query results based on accurate mass 166.0530 with 10 ppm mass accuracy. Three metabolites were retrieved.

| **Total: 3 Metabolites** |
| --- |

| **METLIN ID** | **MASS** | **Δppm** | **NAME** | **MS/MS** | **STRUCTURE** |
| --- | --- | --- | --- | --- | --- |
| [6428](http://metlin.scripps.edu/metabo_info.php?molid=6428) | [M+H]^+^ **m/z** **166.0532** M 165.0460 | 1 | **DL-Methionine sulfoxide** *Formula:* C5H11NO3S *CAS:* 62697-73-8 |  | [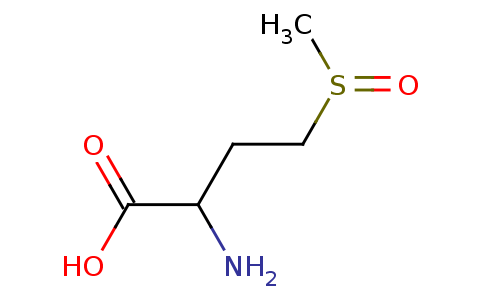](http://metlin.scripps.edu/Mol_images/6428.png) |
| [92035](http://metlin.scripps.edu/metabo_info.php?molid=92035) | [M+H]^+^ **m/z** **166.0532** M 165.0460 | 1 | **Ethiin** *Formula:* C5H11NO3S *CAS:* 17795-08-3 | NO | [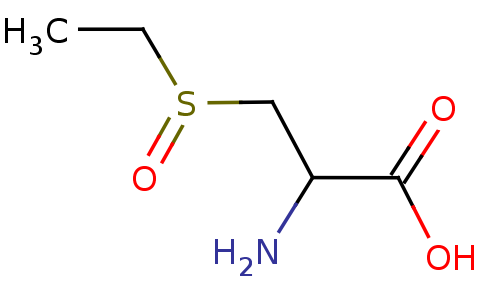](http://metlin.scripps.edu/Mol_images/92035.png) |
| [63430](http://metlin.scripps.edu/metabo_info.php?molid=63430) | [M+H]^+^ **m/z** **166.0532** M 165.0460 | 1 | **L-Methionine S-oxide** *Formula:* C5H11NO3S *CAS:* 3226-65-1 |  | [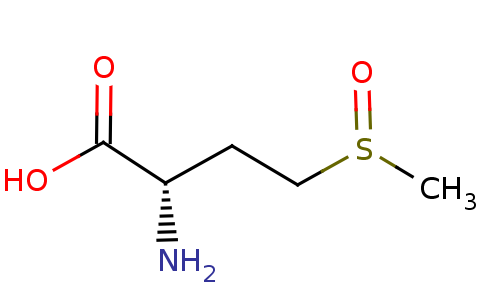](http://metlin.scripps.edu/Mol_images/63430.png) |

**Data S4**: Further evidence shown to support the annotation of peak 434.2175/9.0


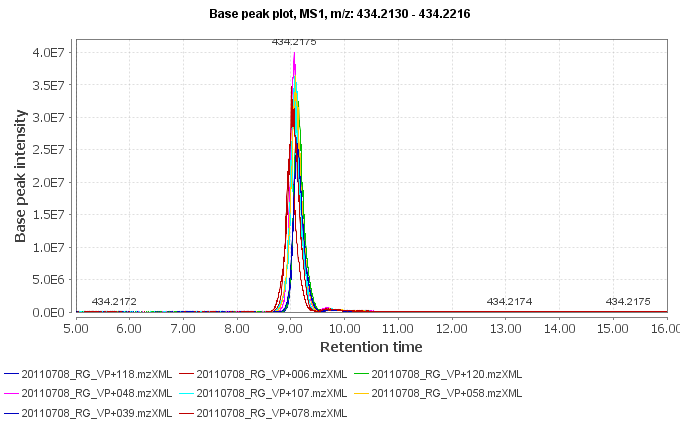


A


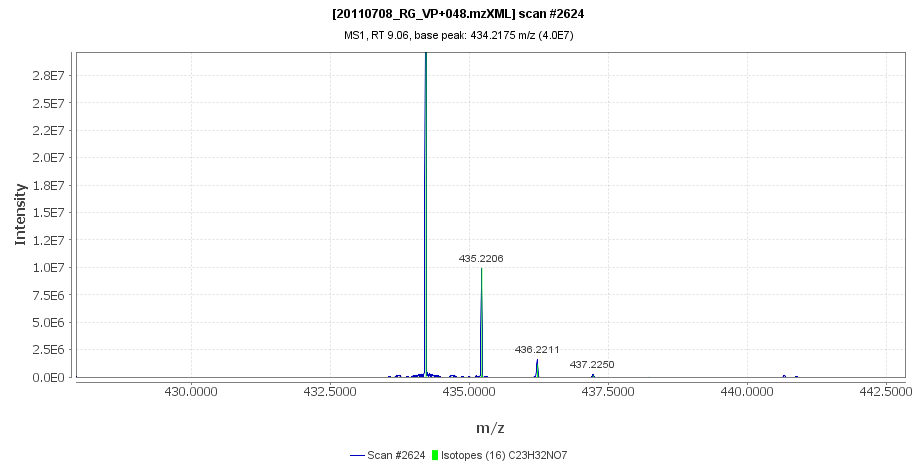


B

C

**Figure S6.** The XIC of *m/z* 434.2173 ([M+H]^+^) eluting at 9.1 min among 8 ryegrass samples (A); and its fitted isotopic ratios (C_23_H_32_NO_7_) using MZmine (one scan, B) and custom R scripts (based on 119 scans, where shown in plot (C) with black bars were averaged relative intensity and green bars were theoretical isotopic distribution of C_23_H_32_NO_7_).


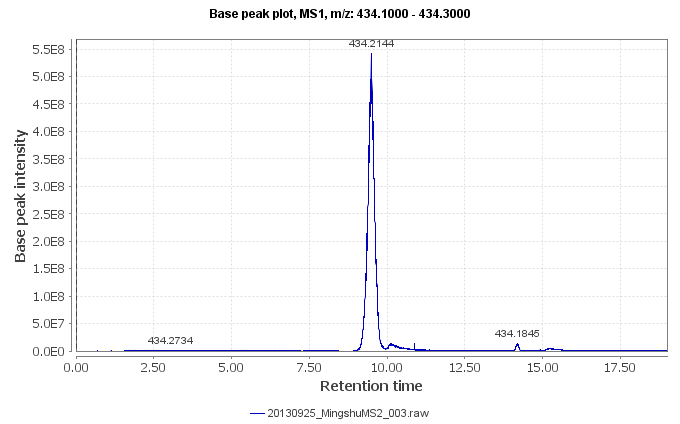


**Figure S7.** Validation of the annotation of *m/z* 434.2173 through the investigation of MS2 fragmentation patterns. XIC of *m/z* 434.1- 434.3 (top) and MS2 of main peak at approximately 9.0 min (bottom).

**Data S5.** Further evidence to support the annotation of peak 132.1023/8.9

**Figure S8**. Extracted ion chromatogram (XIC) of *m/z* 132.1023 of eight samples, overlaid with the two sample groups under either drought stress (red lines) or control condition (blue lines). Peak at 9.57 and 9.86 min can be verified as Leu and Ile, respectively, via spiking experiments. However, the statistically significant peak at 8.89 min remains unknown.

**Figure S9.** Histogram of the predicted retention times (min) of 970 compounds of the same formula C_6_H_13_O_2_ (data source: PubChem)

Among all the compounds there are only 18 compounds that have predicted rts in the range of 8.6 and 9.2 min. The following code snippet reproduces the PubChem IDs of those 23 compounds.

which(pRT>8.6 & pRT < 9.2) –18 compounds

> cid[k3]

[1] "68533" "534604" "575684" "11815876" "12571617" "15508181"

[7] "15788587" "20025860" "20025865" "20235588" "20433152" "21719913"

[13] "21808699" "22047921" "51358378" "53874580" "54165258" "57586949"

**Figure S10.** Validation experiments of Leu through spiking. XIC of 132.10 *m/z* of D-Leucine standard (A); the original ryegrass extract G6-856 (B) and the ryegrass extract spiked with D-Leucine (C). The retention time suggests peak around 10 min (B) can be identified as Leu.

**Figure S11.** Validation experiment of Ile through spiking. Extracted ion chromatograms of 132.10 m/z of isoleucine standard (A), the original ryegrass extract G6-856 (B) and the ryegrass extract spiked with isoleucine (C). The retention time suggests a peak around 10.22 min (B) can be identified as Ile. The validation experiment allows the two adjacent peaks (9.6 and 9.9 min shown in Figure S8) to be identified as Leu and Ile, respectively.
